# Morphological and Molecular Characterization of *Nigrospora oryzae* Causing Leaf Blight and Wilt in *Oreopanax ecuadorensis*

**DOI:** 10.3390/jof12070510

**Published:** 2026-07-10

**Authors:** Daysi Guamán, Carlos Bolaños-Carriel, Nancy Nénger-Coral, Ligia García, Víctor Manuel Valdiviezo Sir, Jaris Veneros

**Affiliations:** 1 Laboratorio de Microbiología y Fitopatología, Facultad de Ciencias Agrícolas, Universidad Central del Ecuador, Av. Universitaria, Tumbaco, Quito 170521, Pichincha, Ecuador; dvguaman@uce.edu.ec (D.G.); cabolanosc@uce.edu.ec (C.B.-C.); nfnenger@uce.edu.ec (N.N.-C.); 2 Instituto de Investigación Para el Desarrollo Sustentable de Ceja de Selva (INDES-CES), Universidad Nacional Toribio Rodríguez de Mendoza de Amazonas, 342 Higos Urco, Chachapoyas 01001, Peru; ligia.garcia@untrm.edu.pe (L.G.); victor.valdiviezo@untrm.edu.pe (V.M.V.S.); 3 Facultad de Ingeniería Zootecnista, Biotecnología, Agronegocios y Ciencia de Datos, Universidad Nacional Toribio Rodríguez de Mendoza de Amazonas, 342 Higos Urco, Chachapoyas 01001, Peru

**Keywords:** leaf blight, phylogenetic analysis, Koch’s postulates, plant–pathogen interaction, endemic species

## Abstract

The genus *Nigrospora* remains poorly understood as a plant pathogen. Until 2026, there were only 26 species recorded in MycoBank and 19 in GenBank. Diseases caused by *Nigrospora* were once considered uncommon, but in recent years there has been a sharp rise in first reports globally, especially in tropical and subtropical regions such as China, Malaysia, India, and the Americas, as well as in Europe. In November 2022, *Oreopanax ecuadorensis*, a plant endemic to Ecuador, exhibited symptoms of leaf blight and wilting in Quito Metropolitan Park-South, Ecuador. The causal agent was isolated on PDA; DNA was extracted, and PCR products of the ITS and EF regions were sequenced. DNA sequences from isolate CBC-FCA-001 were deposited in GenBank under accession numbers OR597663.1 (ITS region) and PP897887.1 (EF region). Morphological and molecular analyses identified the causal agent as *Nigrospora oryzae*. Koch’s postulates confirmed the isolate’s pathogenicity, with mycelium-inoculated, non-wounded plants exhibiting characteristic symptoms while control plants remained healthy. This marks the first report of *N. oryzae* affecting *O. ecuadorensis.* Symptoms were observed on multiple plants throughout the site, raising concerns about the vulnerability of endemic flora to emerging pathogens. The rise in *Nigrospora*-associated diseases, potentially driven by climate change and human activities, highlights the urgent need for research to mitigate their impacts on agriculture and biodiversity conservation.

## 1. Introduction

Deforestation and degradation of native species are critical challenges in Ecuador, threatening biodiversity and essential ecosystem services [1]. Among the affected species is the Pumamaqui, *Oreopanax ecuadorensis* Seem (Araliaceae). Pumamaqui is an endemic species of Ecuador, frequently found in remnant areas of Andean vegetation, along rivers and ravines, within ecological reserves, and in nearby areas of protected National Parks of the Andes of Ecuador [2]. Adapted to mountainous regions, Pumamaqui thrives at altitudes between 1500 and 3500 m above sea level. Despite its ecological importance, the pathology of *O. ecuadorensis* remains poorly studied. While some defoliating insects and fungal pathogens such as Rhizoctonia have been noted to cause mortality in seedlings and young trees [3,4], the range of diseases threatening this endemic species in natural habitats is largely unknown.

Recently, severe symptoms of leaf blight and wilting were observed in *O. ecuadorensis* populations at the Quito Metropolitan Park. Preliminary microscopic observations of the affected tissues suggested the presence of a *Nigrospora* species. Species within the genus *Nigrospora* are increasingly recognized as significant plant pathogens in tropical and subtropical regions, causing foliar spots, necrosis, and plant death across a wide range of hosts [4,5]. However, their impact on native Andean flora has not been assessed. Therefore, the objectives of this study were to characterize the *Nigrospora* isolate associated with the diseased *O. ecuadorensis* using morphological and molecular approaches, and to fulfill Koch’s postulates to confirm its pathogenic effect. This study is the first investigation in Ecuador to examine the impact of a *Nigrospora* species on Pumamaqui, which is critical for maintaining Andean biodiversity and ecosystem stability.

The genus *Nigrospora* Zimm. (*Apiosporaceae*, Xylariales, and Sordariomycetes) encompasses a wide ecological diversity, including species that can be both saprophytic and phytopathogenic. Colony morphology of *Nigrospora* is characterized by rapid growth, producing woolly colonies on potato dextrose agar at 25 °C [6]. Colonies mature within four days, initially appearing white, gradually turning gray with black areas, and eventually becoming entirely black on both the surface and reverse [7]. Sporulation can take over three weeks in isolation.

Microscopically, *Nigrospora* exhibits septate, hyaline hyphae, with hyaline or slightly pigmented conidiophores [8]. Conidiophores bear solitary, black conidia measuring 14–20 µm in diameter, with a thin, equatorial germ slit [9]. These unicellular conidia are slightly flattened horizontally and are borne singly at the tips of the conidiophores [10]. Traditionally, species identification within this genus has relied on morphological methods, focusing on the size and shape of conidia and conidiophores, as well as colony color [11]. However, morphology can be influenced by environmental factors, affecting the stability of these characteristics and leading to intermediate forms, which complicates the precise and reliable differentiation of *Nigrospora* species [12].

Differentiating *Nigrospora* species by host may not be reliable, as taxa such as *N. sphaerica* and *N. oryzae* infect a wide range of host plants [13]. The use of molecular markers in fungal taxonomy has the potential to clarify relationships among taxa that have not been adequately distinguished by morphological studies [14]. These techniques facilitate understanding of the genetic identity and complexity of pathogen populations that affect specific hosts, which is crucial for accurate disease diagnosis [15]. Phylogenetic analyses based on a single gene have not proven highly effective for species delineation. This is partly due to the high rate of nomenclature errors in GenBank, which has caused issues in the taxonomy of *Nigrospora* and complications in identifying specific taxa. Consequently, current recommendations suggest using epitypification or multilocus phylogenies to delineate species and achieve a more accurate understanding of the genus [5].

While the genus *Nigrospora* was historically poorly studied, taxonomic delineation has rapidly advanced in recent years. As of 2025, there are over 26 species recorded in MycoBank and 19 sequences in GenBank [13,15,16]. Leaf spots caused by *Nigrospora* sp. are uncommon diseases that have spiked dramatically in regions such as China [17], Malaysia [18], India [12]. In recent years, the number of reports of *Nigrospora oryzae* causing disease in various hosts has increased notably in the American Phytopathological Society journals’ database (Table A1).

*Nigrospora* species have been recognized as significant plant pathogens, affecting a wide range of hosts across various regions. The completion of the *Nigrospora osmanthi* genome sequence is a significant advancement in understanding the molecular mechanisms driving its pathogenicity [19]. *Nigrospora* species are known to cause leaf spots and blight diseases; however, it is unknown how ecologically important they are to plants. In Korea, *Nigrospora oryzae* was identified as the causative agent of leaf spot on peanut (*Arachis hypogaea*) [20]. Similarly, in China, *N. oryzae* has been associated with leaf spot disease on *Amorphophallus albus*, *Lonicera japonica*, and other hosts [17,21,22]. Another species, *N. sphaerica*, has been reported to induce leaf blight on pumpkin [17] and leaf spot on *Rhododendron simsii* [23] and *Zanthoxylum bungeanum* [24]. In Sichuan, China, the pathogenicity of *Nigrospora musae* was confirmed by its ability to cause stem blight on *Taxus chinensis* var. mairei, a plant of medicinal and ecological importance [25].

*Nigrospora oryzae* is prevalent in tropical and subtropical regions. Humans have a remarkable impact on unique flora and fauna. Intensive agriculture and the introduction of exotic pathogens can lead to the demise of endemic flora. The damage caused by pests and diseases that were not evident in the past but are now causing problems is likely linked to climate change.

Therefore, the objectives of this study were to characterize *Nigrospora oryzae* in *Oreopanax ecuadorensis* and fulfill Koch’s postulates to confirm the pathogenic effect of *Nigrospora oryzae* in *Oreopanax ecuadorensis.* This study is the first investigation in Ecuador to examine the impact of *Nigrospora* sp. on Pumamaqui. The findings enhance understanding of the impact of *Nigrospora* sp. on this native species. Protecting Pumamaqui is critical for maintaining Andean biodiversity and ecosystem stability.

## 2. Materials and Methods

Sampling Material and collection area:

In November 2022, symptoms of leaf blight and wilting were observed at the Quito Metropolitan Park, South (0.3416° S, 78.5211° W), Pichincha province, Ecuador. The symptoms were observed as affecting approximately 30% of the *Oreopanax ecuadorensis* trees in the area. Fresh leaf samples showing symptoms were collected in paper bags for immediate fungal isolation. Additionally, duplicate samples were dried in herbarium presses for voucher deposition. Affected leaves had small, black, irregular-to-circular spots with black-brown pustules and a musty stroma. Microscopic and macroscopic structures were analyzed at the Plant Pathology and Microbiology laboratory of the Universidad Central del Ecuador—Agronomy Faculty, Quito, Ecuador. The statistical analysis and species identification were conducted at the INDES-CES laboratories at UNTRM.

Isolation, purification, and morphological characterization:

The plant material was sterilized using a 1% sodium hypochlorite solution (3 min) and 70% ethanol (1 min) to eliminate surface contaminants. This process was followed by three rinses with sterile distilled water to remove any residual disinfectant and ensure a clean surface. Once disinfected, small fragments of the plant material were aseptically excised using a sterile scalpel. These fragments were dried on a sterile paper towel, then transferred to potato dextrose agar (PDA) and Sabouraud Dextrose Agar (SDA) culture medium under aseptic conditions in a laminar flow cabinet. Samples were incubated in a laboratory incubator at 25 °C for one week.

Using a sterile cork borer, a segment of mycelium from a selected colony was carefully extracted and transferred to a Petri dish containing PDA medium. This procedure was repeated two to four times to ensure the recovery of pure isolates. Purity was confirmed after seven days of incubation at 25 °C, as evidenced by uniform colony growth free from contaminants. Once pure isolates were obtained, taxonomic identification was performed. This process involved both macroscopic characterization of the colonies and microscopic observation of reproductive structures, following established methods [26]. Macroscopic characterization focused on observing colony features, including color, shape, texture, and mycelial elevation. Microscopic characterization was conducted by observing reproductive structures using the slide-culture method [27]. A portion of mycelium from a pure isolate was transferred to two PDA blocks measuring approximately 1 cm^2^. These blocks were placed on a sterile glass slide supported by two sterile wooden sticks and a layer of filter paper. The inoculated blocks were covered with a sterile cover slip, and 2 mL of sterile distilled water was added to create a humid chamber. The samples were incubated at 25 °C for seven days. After incubation, the cover slip was carefully removed, and the structures were stained with lactophenol blue for observation under an inverted microscope, OLYMPUS (New York Microscope Company, Hicksville, NY, USA), at 40× magnification. The morphometric analysis of reproductive structures, particularly conidia, was performed by measuring their dimensions (µm) using the IMAGE J v. 1.5.3 software developed by Wayne Rasband at the National Institutes of Health (NIH) [28]. Photomicrographs of observed structures were captured using a TECNO SPARK 10 PRO digital camera mounted on the inverted microscope (OLYMPUS, Tokyo, Japan). Images were uploaded to IMAGE J for measurement of the length and width of 30 conidia from the purified isolate. The software’s scale was calibrated using a Neubauer chamber photograph at 40× magnification. This calibration ensured that measurements were automatically expressed in micrometers (µm) [29]. For conidial measurements, length was determined perpendicular to the widest part of the structure, while the width was measured at the broadest section. Following purity confirmation, ten fungal isolates displaying similar morphological characteristics were obtained and designated as CBC-FCA-001 through CBC-FCA-010. These isolates were preserved for further analysis.

Molecular characterization:

The molecular analysis began with DNA extraction and amplification performed at the Laboratory of Plant Genetics at the Faculty of Agricultural Sciences, Universidad Central del Ecuador. DNA was extracted from pure isolates using the Total Genomic DNA Isolation kit (Norgen Biotek Corp., Thorold, ON, Canada) according to the manufacturer’s protocol. DNA was extracted from ten isolates (CBC-FCA-001 to CBC-FCA-010). For each sample, 150 milligrams of mycelium were weighed and macerated in a mortar with 1 mL of lysis buffer to create a homogeneous mixture. This mixture was transferred to sterilized microcentrifuge tubes, incubated at 65 °C for 10 min with intermittent shaking, and then centrifuged at 14,000 rpm for 2 min. The supernatant was carefully transferred to a new tube, ethanol was added in equal volume, and subsequent steps were performed according to the kit’s protocol to purify the DNA using spin column filtration and elution with Elution Buffer. Purified nucleic acids were stored at −40 °C for further use.

DNA purity and concentration were quantified using a Nanodrop 2000 spectrophotometer (Thermo Fisher Scientific, Asheville, NC, USA). PCR amplification was conducted using Platinum SuperFi Master Mix Invitrogen (Thermo Fisher Scientific, Asheville, NC, USA) in 25 µL reaction volumes. The reactions included 12.5 µL of Master Mix, 1.25 µL of each primer, 3 µL of sterile distilled water, 5 µL of SuperFi GC Enhancer, and 2 µL of DNA template. A portion of the ITS1, 5.8 ribosomal gene, and ITS2 gene was amplified using primers ITS-1 (5′-TCCGTAGGTGAACCTGCGG-3′) and ITS-4 (5′-TCCTCCGCTTATTGATATGC-3′) [22] and sequenced. An overlapping fragment of approximately 1000 bp that extends nearly to the end of EF1-a was amplified with primers 983F (5′-GCYCCYGGHCAYCGTGAYTTYAT) and 2218R (5′-ATGACACCRACRGCRACRGTYTG) [23]. PCR consisted of an initial denaturation at 95 °C for 3 min, followed by 35 cycles of denaturation (95 °C, 30 s), annealing (54 °C, 30 s), and extension (72 °C, 60 s), with a final extension at 72 °C for 45 s. Agarose gel electrophoresis for DNA analysis followed standardized procedures. Agarose (0.4 g) was dissolved in 40 mL of 1X TBE buffer by heating in a microwave. Once cooled to a tolerable temperature, 1.5 µL of SYBR were safely added to the mixture, which was poured into a mold with a comb to create wells. After solidification, the gel was submerged in TBE buffer, and DNA samples mixed with loading dye were loaded into the wells alongside a 100 bp DNA ladder. The electrophoresis system was run at 140 V for 30 min, and the resulting bands were visualized using a biorad photo-documentation system. For further refinement, PCR products were purified using the PureLink PCR Purification Kit (Invitrogen, Carlsbad, CA, USA) and prepared for sequencing.

The purified DNA fragments were sequenced at Macrogen (Rockville, MD, USA). Sequences were trimmed in CodonCode Aligner, and BLAST search was performed at NCBI to find matching sequences and confirm preliminary species identification. Phylogenetic analysis was then performed to determine the taxonomic placement of the isolates. For the phylogenetic analysis, reference ITS sequences of representative *Nigrospora* species were retrieved from GenBank. Sequences derived from type specimens (ex-type, epitype, or neotype) were prioritized and included to ensure accurate species delineation, along with sequences of *N. oryzae* and other closely related taxa. The retrieved sequences were aligned using MEGA 11. The JModelTest program was used to select the most appropriate evolutionary model. Sequences were then formatted into NEXUS files using Mesquite and analyzed with MrBayes 3.2.6 employing the Markov Chain Monte Carlo (MCMC) algorithm. Bayesian phylogenetic trees were generated, and a maximum clade credibility (MCC) tree was constructed using FigTree version 1.4.2.

Pathogenicity testing:

Inocula of two representative *Nigrospora* isolates (CBC-FCA-001 and CBC-FCA-002) were obtained from PDA plates incubated for 15 days at 22 °C in the dark. Pathogenicity was evaluated using two complementary assays: a detached-leaf assay to rapidly confirm foliar symptom development and a whole-plant assay to assess systemic effects, such as wilting. For the detached leaf assay, healthy leaves of *O. ecuadorensis* were superficially disinfected with 1% sodium hypochlorite for 3 min, then rinsed with sterile water for 5 min. Agar discs (6 mm diameter) with mycelium were taken from the edge of the growing colony and placed mycelium side down on the central vein of the underside of the leaves. A negative control with sterile agar discs was included. The inoculated leaves were placed in plastic trays with a thin film of sterile distilled water at the bottom, hermetically sealed to create a moist chamber, and maintained at room temperature (24 °C).

For the whole plant assay, three *O. ecuadorensis* plants (1.3–1.5 m tall) were inoculated using the same mycelial agar discs placed on the apical tissue, while an additional plant was left uninoculated as a control. Plants were kept in a greenhouse for three weeks. The re-isolated fungus was identified as *Nigrospora oryzae* based on its characteristic morphological features, thus fulfilling Koch’s postulates.

## 3. Results

**Figure 1:**
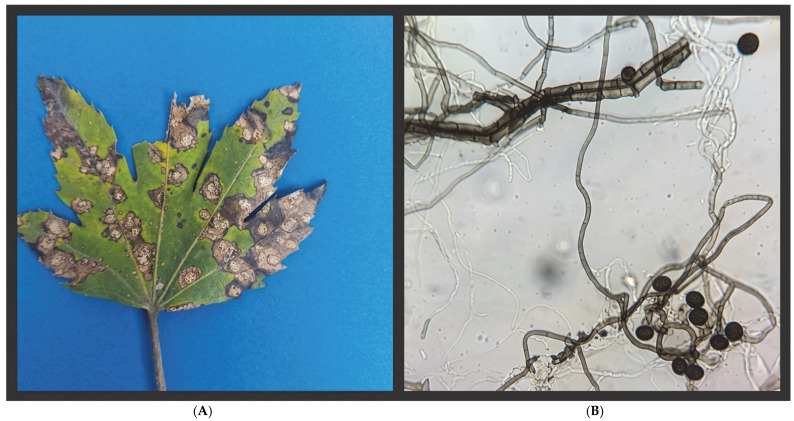
Symptoms and fungal structures of *Nigrospora* sp. causing leaf blight in *Oreopanax ecuadorensis* (**A**) leaf blight symptoms; (**B**) light microscopy of isolates of *Nigrospora* sp. at 40×.

Ten fungal isolates were obtained from leaves of Pumamaqui plants. The *Nigrospora* sp. isolate was cultured on PDA medium and incubated at 25 °C in the dark for 7 days. On PDA, colonies were circular and white, turning gray and dark with age. The fungus sporulated after three weeks at 25 °C. After the incubation period, colonies exhibited rapid growth, initially appearing white and later transitioning to gray and black due to abundant sporulation. The colonies displayed the following morphological characteristics: filamentous shape, wrinkled elevation, filamentous edges, radial surface pattern, and abundant aerial mycelium. The colony’s upper surface was black, while the underside appeared grayish white (Figure 1).

**Figure 2:**
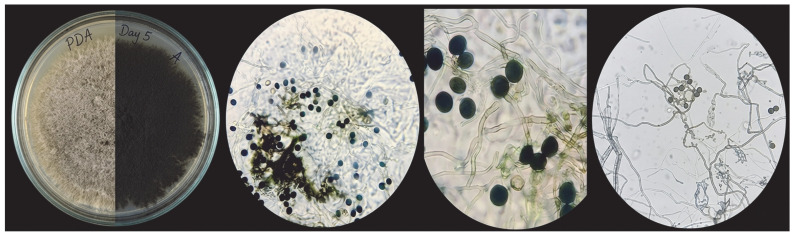
Colony on PDA medium after 7 days, showing conidia and hyphae observed under a light microscope at 40× magnification for *Nigrospora oryzae* isolated from *Oreopanax ecuadorensis*.

Light microscopy examinations of pure cultures obtained using the single-spore method showed black sub-epidermal sori with no paraphysis. Mycelia were filamentous, and melanized (dematiaceous) hyphae were septate. Fruiting bodies were individually spherical to oblong, and conidia were black and measured (9.0–13.2) × (12.6–15.8) μm (n = 20). The conidia were identical to the textbook description [10] (Figure 2).

Sequences of Isolate CBC-FCA-001 were deposited in GenBank under the accession Numbers OR597663.1 (465 nt from the ITS region) and PP897887.1 (922 nt from the Elongation Factor region). Based on these molecular results, the isolate previously identified morphologically as a *Nigrospora* sp. was confirmed as *Nigrospora oryzae*, as the sequences shared 99.8% and 96% identity with *N. oryzae* sequences MT150620.1 and CP096804.1, respectively.

Leaves of *Oreopanax ecuadorensis* inoculated with agar discs containing mycelium began to exhibit black spot symptoms around the agar discs after seven days. This phenomenon suggests the initial colonization of the fungus in the leaf tissue. The subsequent appearance of black pustules in the affected areas confirmed the sporulation of *Nigrospora oryzae*. This ability to produce dark conidia in pustular structures is a classic marker of fungal proliferation in infected leaves and pathogenic aggressiveness.

**Figure 3:**
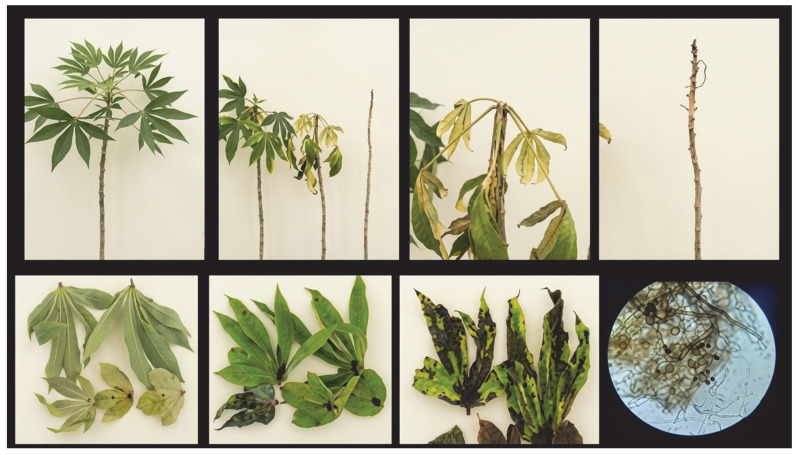
Koch’s postulates to prove the pathogenicity of *Nigrospora oryzae* on *Oreopanax ecuadorensis*: Three plants with intact tissue were inoculated, and the fourth plant was inoculated with water. The fungus was inoculated on detached leaves. Symptoms were observed on days 5 and 15. Isolation from infected leaves produced colonies of *N. oryzae* on PDA medium after 7 days.

Over time, the necrosis spread across the leaf, indicating progressive damage to the foliar tissue (Figure 3). *Nigrospora oryzae* was re-isolated from the symptomatic leaves and cultured on PDA medium. The consistency of these morphological features confirmed that the observed symptoms were caused by the fungus, supporting its identification as the causal agent of the infection. The absence of such symptoms in control leaves inoculated with agar discs lacking mycelium confirms *N. oryzae* as the causative agent.

Koch’s postulates showed that initial symptoms appeared 15 days after inoculation. The leaves turned completely black and wilted. For fully grown plants, wilting of inoculated tissue with a black and musty mass of mycelia was observed, as well as the collapse of one inoculated plant. The check plant did not collapse or present wilting (Figure 3).

**Figure 4:**
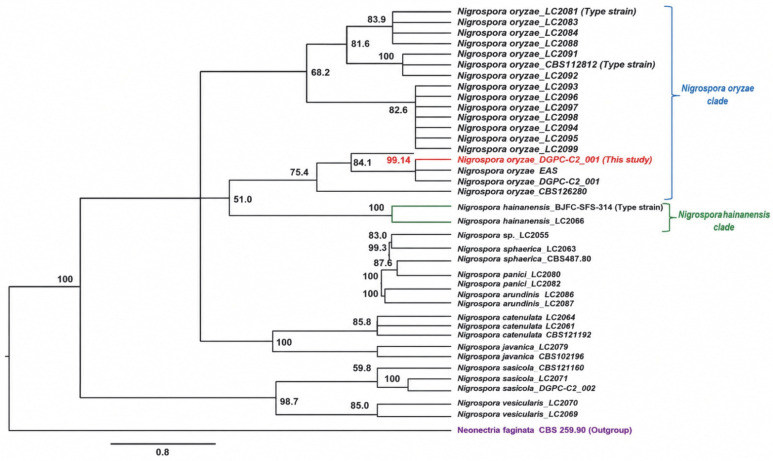
Phylogenetic tree of the genus *Nigrospora* based on ITS sequences, showing the placement of isolate CBC-FCA-001 within the genus. Species-level confirmation was achieved via EF sequencing.

A phylogenetic analysis was performed to determine the taxonomic placement of the isolate. Initially, an ITS tree was constructed using 54 sequences of the genus *Nigrospora*, with *Apiospora malaysiana* (CBS 102053) as the outgroup. Bayesian analysis (using the TrNef + G model and MCMC algorithm in MrBayes) placed the isolate within the *Nigrospora* clade. However, because the ITS region alone cannot reliably distinguish *N. oryzae* from closely related species such as *N. hainanensis* and *N. rubi*, species-level identification relied on the Elongation Factor (EF) region. BLAST analysis of the EF sequence (PP897887.1) revealed a high identity with confirmed *N. oryzae* sequences. Phylogenetic inference based on the EF region confirmed that the isolated sample belongs to the *Nigrospora oryzae* branch, which is clearly separate from other species complexes within the genus (Figure 4).

## 4. Discussion

Leaves of *Oreopanax ecuadorensis* with small black and irregular to circular spots with black-brown pustules and musty stroma were observed in Quito, Ecuador. Affected areas of the leaves can become chlorotic, and this discoloration may spread across the entire leaf [11,30]. Spots can lead to necrosis of the leaf tissue, ultimately resulting in the death of the affected areas [31]. *Nigrospora oryzae* is considered the plant pathogen associated with Pumamaqui. It has been reported that *Nigrospora oryzae* causes leaf spot disease on *Chrysanthemum × morifolium Ramat*, with dark brown spots and small, irregular points on the leaves, noting that individual spots can merge to form large, irregular blotches [32]. The sequence deposited in GenBank (accession number PP897887.1), corresponding to *Nigrospora*, was analyzed, specifically the elongation factor (EF) gene region, which is 922 nucleotides (nt) long. This sequence was identified as *Nigrospora* UCE-FCA_EF_F1983F.ab1.

A comparison of sequence PP897887.1 with other sequences in the GenBank database revealed high similarity to two *Nigrospora oryzae* sequences. Specifically, it exhibited 97.68% similarity to sequence PP761001.1. This high similarity suggests that PP897887.1 is nearly identical to this particular *Nigrospora oryzae* sequence, with the 2.32% difference likely attributable to minor variations or point mutations. Additionally, its 96% similarity to CP096804.1, although slightly lower, still indicates a close phylogenetic relationship with *Nigrospora oryzae*, reflecting potential intraspecific variations or distinct strains within the species. The percentage of similarity suggests a reliable preliminary identification but also raises the possibility of intraspecific variability or divergent lineages [32].

Liu et al., 2024 [13], reported that *Nigrospora oryzae* can act as an opportunistic pathogen in rice plants, underscoring the importance of accurate identification of this species, particularly in agricultural contexts. However, additional research has shown that some strains of *Nigrospora oryzae* exhibit beneficial endophytic functions, including the production of antimicrobial secondary metabolites [33]. Phylogenetic studies of *Nigrospora* isolated from various geographic regions have reported significant genetic divergence, with some populations exhibiting less than 95% similarity across molecular markers [4,34]. This suggests that diversity within the genus may be driven by local speciation processes. Such findings could be relevant for interpreting the 98% similarity observed in our analysis, particularly since our sample was obtained from a unique environment involving an endemic host like Pumamaqui.

The aligned and trimmed ITS and EF sequences, when entered into the BLAST program, exhibited >99% identity to *Nigrospora oryzae*, thereby confirming the initial morphological identification. Seventeen clades corresponding to *Nigrospora* species were identified, consistent with previous studies by Sha et al. (2023) [32] and Wang et al. (2017) [4], who used a similar methodology and identified 18 clades, confirming the robustness of phylogenetic analyses based on ITS genes and Bayesian probabilities.

To our knowledge, this is the first report of *Nigrospora oryzae* causing leaf blight and musty wilt of *Oreophanax ecuadoriensis*. Despite the sudden increase in *Nigrospora* and initial reports on the causes of plant diseases worldwide, much about the biology and ecology of the genus remains unknown, and further research is needed to fully understand the role of these fungi as plant pathogens.

### Limitations and Prospects

This study has some limitations that need to be acknowledged. Firstly, while the combination of ITS and EF markers successfully identified the pathogen, a more comprehensive multilocus phylogenetic analysis including additional markers such as TUB2 and RPB2, alongside sequences from type specimens, would provide definitive resolution within the *Nigrospora* genus. Our identification is based on the high similarity of the EF region to ex-type sequences and congruent morphology; however, future studies should prioritize whole-genome sequencing or multilocus approaches to elucidate the precise phylogenetic boundaries of *Nigrospora* species in Ecuador. Future research should focus on multilocus phylogenetic analyses and genomic approaches to more accurately define species boundaries within the genus *Nigrospora*. Additionally, epidemiological studies are necessary to evaluate the distribution, incidence, and severity of this pathogen in natural populations of *Oreopanax ecuadorensis*. It is crucial to understand how environmental factors, particularly climate change, contribute to the emergence of diseases. Furthermore, additional studies should explore *Nigrospora*’s dual ecological role as an endophyte and a pathogen, as well as potential management strategies to mitigate its impact on endemic Andean flora.

## Data Availability

The original contributions presented in this study are included in the article. Further inquiries can be directed to the corresponding author.

## Appendix A

**Table A1:** Database of recent reports of *Nigrospora oryzae* as a plant pathogen.

Authors	Title
Sang-Min Kim, A9:A18, Shinhwa Kim, Jieun Lee, Soo Yeon Choi, Hyunjung Chung, JaeBuhm Chun, Bo Yoon Seo, Ju-Rak Lim, and Nak jung Choi	First Report of *Nigrospora oryzae* Causing Leaf Spot on Peanut (Arachishypogaea) in the Republic of Korea (https://apsjournals.apsnet.org/doi/10.1094/PDIS-05-24-0954-PDN (accessed on 1 June 2026))
L. F. Duan, C. Shen, Y. X. Song, G. X. Zhou, J. F. Qin, Y. G. Cai, Y. Y. Zhang, and B. F. Qin	First Report of *Nigrospora oryzae* Causing Leaf Spot Disease on Amorphophallus albus in Shaanxi Province of China (https://apsjournals.apsnet.org/doi/10.1094/PDIS-04-24-0767-PDN (accessed on 1 June 2026))
Xue Chen, Hui Chang, Yanlei Li, Yanan Lin, Hongyan Liu, Yongqing Zhang, Yanhong Bai, Fengxia Han, and Qian Liu	First Report of Leaf Spot Disease on *Lonicera japonica* Caused by *Nigrospora oryzae* in China (https://apsjournals.apsnet.org/doi/10.1094/PDIS-01-24-0190-PDN (accessed on 1 June 2026))
Xinyu Lu, Chun Xiao, Kangxu Zhou, Linghui Fu, Danyu Shen, and Daolong Dou	First Report of *Nigrospora oryzae* Causing Leaf Spot on Yam in China (https://apsjournals.apsnet.org/doi/10.1094/PDIS-11-22-2545-PDN (accessed on 1 June 2026))
Guangling Xu, Tiantian Yang, Fengxia Tian, and Tan Wang	Leaf Spot Disease on Euonymus japonicus Caused by *Nigrospora oryzae* in China (https://apsjournals.apsnet.org/doi/10.1094/PDIS-05-22-1188-PDN (accessed on 1 June 2026))
